# Supplementary material for: Placental Cyclophilin A Expression in Pregnancies Complicated with Hypertension
Source: Int J Environ Res Public Health. 2022 Apr 29;19(9):5448. doi: 10.3390/ijerph19095448 (PMC9101619; doi:10.3390/ijerph19095448)
Supplement: Supplementary file 1 [file ijerph-19-05448-s001.zip › ijerph-1646893-supplementary.pdf]

Supplementary Table S1. Expression of Cyclophilin A in the placenta of pregnancy with and without hypertension

				Intensity of Cyclophilin A expression					Histological changes associated with hypertension		
No	Hypertension	Gestational age	Type of delivery	FEC	CT	ST	MEC	DC	AA	Retention of smooth muscle	Increased SK
1	yes	37w	EMLSCS	2	2	0	3	3	yes	yes	yes
2	yes	32w5d	EMLSCS	3	3	0	3	3	yes	no	yes
3	yes	28w	EMLSCS	2	2	0	3	3	yes	no	yes
4	yes	40w3d	EMLSCS	3	3	0	3	3	no	no	yes
5	yes	35w5d	EMLSCS	3	3	0	3	3	no	no	no
6	yes	37w6d	SVD	3	3	0	3	3	no	no	yes
7	yes	30w2d	EMLSCS	3	3	0	3	3	no	no	yes
8	yes	38w4d	EMLSCS	3	3	0	3	3	no	no	no
9	yes	31w	EMLSCS	1	1	0	3	3	no	no	yes
10	yes	39w5d	EMLSCS	2	2	0	3	3	no	no	no
11	yes	39w1d	EMLSCS	2	2	0	3	3	no	no	no
12	yes	36w2d	EMLSCS	2	2	0	3	3	yes	no	yes
13	yes	37w6d	EMLSCS	3	3	0	3	3	no	no	no
14	yes	32w2d	EMLSCS	2	2	0	3	3	no	no	yes
15	yes	32w	EMLSCS	3	3	0	3	3	no	no	no
16	yes	32w	ELLSCS	1	1	0	3	3	no	no	no
17	yes	34w5d	EMLSCS	2	2	0	3	3	no	no	no
18	yes	36w	EMLSCS	2	2	0	3	3	no	no	yes
19	yes	35w6d	EMLSCS	2	2	0	3	3	yes	yes	yes
20	yes	40w1d	SVD	2	2	0	3	3	yes	no	no
21	yes	26w4d	ELLSCS	2	2	0	2	3	yes	yes	yes
22	yes	36w3d	EMLSCS	1	1	0	3	3	no	no	yes
23	yes	38w2d	SVD	2	2	0	3	3	no	no	no
24	yes	39w2d	SVD	3	3	0	3	3	no	no	no
25	yes	37w4d	EMLSCS	3	3	0	NA	3	no	yes	yes
26	yes	37w4d	SVD	3	3	0	NA	3	no	no	yes
27	yes	39w5d	EMLSCS	3	3	0	3	3	no	no	no
28	yes	26w1d	EMLSCS	3	3	0	2	3	no	no	yes
29	yes	33w	EMLSCS	2	2	0	2	3	yes	yes	yes
30	yes	39w	EMLSCS	3	3	0	3	3	no	no	no
31	yes	38w2d	EMLSCS	3	3	0	3	3	no	no	yes
32	yes	34w	EMLSCS	3	3	0	3	3	no	no	yes
33	yes	29w3d	EMLSCS	3	3	0	3	3	yes	yes	yes
34	yes	30w2d	EMLSCS	3	3	0	NA	3	yes	yes	yes
35	yes	35w3d	EMLSCS	2	2	0	1	3	no	no	yes
36	no	39w1d	SVD	3	3	0	3	3	no	no	no
37	no	38w4d	ELLSCS	3	3	0	NA	3	no	no	no
38	no	37w	SVD	2	2	0	0	3	no	no	no
39	no	37w	SVD	1	1	0	3	3	no	no	no

40	no	38w4d	SVD	3	3	0	3	3	no	no	no
41	no	38w	SVD	3	3	0	3	3	no	no	no
42	no	39w	SVD	1	1	0	2	3	no	no	no
43	no	39w4d	SVD	2	2	0	3	3	no	no	no
44	no	39w4d	SVD	1	1	0	1	3	no	no	no
45	no	39w1d	SVD	2	2	0	3	3	no	no	no
46	no	40w2d	SVD	1	1	0	3	3	no	no	no
47	no	39w	SVD	1	1	0	2	3	no	no	no
48	no	37w	SVD	2	2	0	3	3	no	no	no
49	no	37w4d	SVD	2	2	0	3	3	no	no	no
50	no	37w5d	SVD	1	1	0	3	3	no	no	no
51	no	38w2d	SVD	1	1	0	NA	3	no	no	no
52	no	37w2d	SVD	2	2	0	3	3	no	no	no
53	no	40w1d	SVD	2	2	0	1	3	no	no	no
54	no	39w1d	SVD	2	2	0	NA	3	no	no	no
55	no	40w5d	IAD	2	2	0	3	3	no	no	no
56	no	39w2d	SVD	2	2	0	3	3	no	no	no
57	no	40w9d	EMLSCS	3	3	0	3	3	no	no	no
58	no	38w	SVD	3	3	0	3	3	no	no	no
59	no	40w1d	SVD	2	2	0	3	3	no	no	no
60	no	39w	SVD	2	2	0	NS	3	no	no	no
61	no	39w5d	EMLSCS	2	2	0	NA	3	no	no	no
62	no	39w3d	SVD	2	2	0	NA	3	no	no	no
63	no	36w5d	EMLSCS	2	2	0	3	3	no	no	no
64	no	40w4d	SVD	3	3	0	3	3	no	no	no
65	no	38w	SVD	3	3	0	3	3	no	no	no
66	no	38w	ELLSCS	3	3	0	3	3	no	no	no
67	no	39w2d	EMLSCS	3	3	0	NA	3	no	no	no
68	no	37w5d	SVD	2	2	0	3	3	no	no	no
69	no	38w1d	SVD	2	2	0	3	3	no	no	no
70	no	40w5d	SVD	2	2	0	NA	3	no	no	no

w – week, d – day, SVD – spontaneous vaginal delivery, EMLSCS - emergency lower segment caesarean section, ELLSCS – elective lower segment caesarean section, IAD – instrumental assisted delivery, NA – not available, AA – acute atherosis, SK = syncytial knot, FEC – fetal endothelial cells, CT – cytotrophoblasts, ST – syncytiotrophoblasts, MEC – maternal endothelial cells, DC – decidual cells
